# Are marked body shape concerns associated with poorer outcomes at the one‐year follow‐up in anorexia nervosa?

**DOI:** 10.1002/brb3.2199

**Published:** 2021-05-26

**Authors:** Morgane Rousselet, Hélène Reinhardt, Bastien Forestier, Emeline Eyzop, Sylvain Lambert, Bruno Rocher, Lucie Gailledrat, Jean‐Benoit Hardouin, Marie Grall‐Bronnec

**Affiliations:** ^1^ Addictology and Psychiatry Department Nantes University Hospital Nantes France; ^2^ UMR 1246 Nantes University and Tours University Nantes France

**Keywords:** Anorexia nervosa, body shape concerns, Morgan and Russell, clinical outcome, follow‐up

## Abstract

**Objective:**

Anorexia nervosa (AN) is a complex disease in which obsessive thoughts about body image, shape, or weight are expressed. The intensity of these concerns varies among individuals, and only a few studies have focused on their impact on patients’ clinical course when patients are treated on an outpatient basis. Our study aimed to determine whether marked body concerns at inclusion were predictive of the one‐year follow‐up.

**Method:**

Participants (*N* **= **72) were women seeking treatment for AN in a specialized unit for eating disorder management. All participants were assessed at inclusion and at the 1‐year follow‐up. Clinical outcome was assessed using the Morgan & Russel Outcome Average Score (MROAS), and body concerns were assessed using the Body Shape Questionnaires (BSQ).

**Results:**

Marked body concerns (BSQ score >140) at inclusion were associated with a poorer outcome at the 12‐month follow‐up (lower MROAS “total score”). Other characteristics at inclusion that were predictive of a poorer outcome at 12 months were as follows: higher severity of ED at inclusion, longer hospitalization during follow‐up, and experiencing a lower impact of the illness on school/work life.

**Discussion:**

The results confirmed the importance of a multifocal treatment that should address body concerns and motivation to change. Our results also highlighted the necessity of promoting the maintenance of school/work during the treatment course.

## Graphical abstract

Marked body concerns were associated to poorer outcome after one year of treatment. Family support seemed to play an important role in achieving better outcomes. Impact of AN on schooling/working life played a positive role in clinical course.

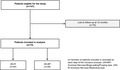

## 1 INTRODUCTION

Anorexia nervosa (AN) is a frequently occurring eating disorder (ED) that mainly affects young women. Despite the progress that has been made in understanding and supporting this condition, its prognosis remains unclear. According to the American Psychiatric Association, AN has a prevalence of 0.4%, and the mortality is evaluated to be approximately 5% per decade, with death by suicide representing half of this rate and death by the consequences of starvation representing the other half (American Psychiatric Association, 2013). Numerous studies have analyzed the recovery rate of anorexia, and it is usually found that only 30 to 50% of patients recover (Herpertz‐Dahlmann et al., 2001; Stockford et al., 2019; Zipfel et al., 2000).

Body issues play a central role in the symptoms of AN as well as in the psychopathology of the disease (Calugi & Dalle Grave, 2019; Calugi et al., 2018; Mountford et al., 2015; Phillipou et al., 2018). The concept of body image is based on two key elements: a mental picture of one's physical body (including its size, shape, and appearance) and one's attitude toward the physical self (such as thoughts, feelings, and beliefs about one's body Fairburn, 2008; Gailledrat et al., 2016). Body image issues in AN involved several components: weight and shape over‐evaluation, preoccupation, dissatisfaction, and fear of weight gain (Linardon et al., 2018).

Previous literature on neuroimaging studies, neurocognitive data, and self‐report measures has shown that AN is associated with disturbances of self‐perception and body size estimation (Esposito et al., 2016; Kazén et al., 2019; Purcell et al., 2018). Indeed, in contrast to healthy participants, AN patients tended to overestimate body size when pictures of their own body were introduced during neurocognitive tasks. This was not the case for estimation with pictures of other's bodies (Kazén et al., 2019). Another task in which participants had to judge whether or not an aperture was wide enough for them to pass through showed that, contrary to healthy controls, AN patients tended to perceive their own body larger than it was (Guardia et al., 2010). Several hypotheses have been proposed, including the difficulties of the central nervous system in updating the new body (the one with AN) (Guardia et al., 2012), alteration of tactile perception (Crucianelli et al., 2016; Keizer et al., 2012), or impairments in multisensory integration (Gaudio et al., 2014). A recent study showed a tactile perceptual deficit correlated with clinical scores in AN but no impairment in the integration of tactile and visual information (Risso et al., 2020). Consequently, to disturbances in body representation, patients with AN have a higher level of body dissatisfaction (discrepancy between the actual and ideal selves) and more negative thoughts and feelings about their own body. They have a tendency to have a negative interpretation bias of body‐related information that leads to dysfunctional cognitions and behaviors (Brockmeyer et al., 2018). These concerns might reinforce the fear of gaining weight or becoming fat and occupy a significant space in the clinical symptoms of AN (Treasure et al., 2010). Obsessions could lead to excessive checking behaviors or, in contrast, to body measure avoidance (Marzola et al., 2020). Thus, concerns about weight and body shape are considered to be diagnostic criteria for AN in the DSM‐5 (American Psychiatric Association, 2013; J. F. Morgan et al., 2014). These concerns represent a complex symptom that can fluctuate throughout the course of ED and that may also vary widely among patients in both their intensity and their specificities (Skrzypek et al., 2001). These disturbances can affect the size, weight, shape, or volume of the body and are usually focused on specific parts, most commonly, the abdomen (Keizer et al., 2012), buttocks, or thighs (Kachani et al., 2013). The amount of importance the patient places on the discrepancy between the ideal and the actual self is also a factor to consider (Lantz et al., 2018) as well as negative mood that has also been shown to increase body size perception (Hepworth et al., 2010; Taylor & Cooper, 1992; Tuschen‐Caffier et al., 2015). A previous study carried out in our department showed that the intensity of body shape concerns at the beginning of treatment for ED was linked to specific psychopathological profiles and could be associated with the severity of the disease (Gailledrat et al., 2016).

Body shape concerns are a relevant factor to consider, as they could be involved in the etiology and persistence of AN (Ricca et al., 2010) may be a risk factor for relapse (Keel et al., 2005) and is likely a predictor of poor treatment outcomes (Boehm et al., 2016; Calugi et al., 2018; Vall & Wade, 2015). To date, studies that have identified body shape and weight concerns as predictors of outcomes for AN were carried out in inpatient settings (Boehm et al., 2016; Calugi & Dalle Grave, 2019; Fennig et al., 2017; Marzola et al., 2020; Vall & Wade, 2015; Wales et al., 2016), although inpatient treatments are not the reference treatment for AN (National Collaborating Centre for Mental Health (UK), 2004). Two studies with the aim of assessing specific interventions for AN treatment (cognitive–behavioral therapy (CBT), focal psychodynamic psychotherapy (FPT)) in an outpatient setting showed mixed results. The first one showed no direct prediction of body image disturbance on the outcome of AN treatment but body image disturbance seemed to play an indirect role *via* perceived stress and depressive symptoms (Junne et al., 2019). The second one demonstrated that AN patients with higher shape concerns had poorer outcomes when treated with CBT (Ricca et al., 2010).

The aim of our study was not to examine a specific treatment outcome but to determine whether marked body concerns at inclusion were predictive of the outcome at the one‐year follow‐up when patients with AN were treated as usual (in an outpatient basis according to the guidelines for ED management). The results of this study may aid in the development of new strategies for treatment and relapse prevention.

## 2 METHODS

### 2.1 Procedure and ethics

Since September 2012, an in‐depth clinical assessment has been carried out systematically for all new ED patients referred to our unit for treatment. The aforementioned assessment, which is part of the EVALuation of behavioral ADDictions (EVALADD) cohort (NCT01248767), occurs prior to the first medical consultation (at inclusion) and is then re‐administered at predefined intervals (at 6 months, 12 months, and then every year). This assessment aims to highlight the risk factors involved in ED initiation and persistence. The main criteria for inclusion in the cohort are as follows: age of 15 years or older and a diagnosis of ED as defined by the DSM. Patients with cognitive impairment or difficulties reading or writing French were not included. All patients participate in a face‐to‐face semi‐structured interview and complete self‐report questionnaires. Qualified and experienced staff members perform these assessments. For this specific study, we considered only data from the inclusion assessment and the 12‐month follow‐up assessment.

The EVALADD cohort was conducted in accordance with Good Clinical Practice Guidelines and the Declaration of Helsinki, with approval from the local ethics committee (Groupe Nantais d’Ethique dans le Domaine de la Santé, GNEDS, Nantes‐ Number 2012–09–06). All participants provided written informed consent, including consent from parents or guardians for the participants under age 18. No compensation was given for participation.

### 2.2 Participants

The participants were patients of the EVALADD cohort. For this specific study, data were collected between September 2012 and December 2016. The specific inclusion criteria were as follows: (i) female patients, (ii) an AN restricting (AN‐R) or binge‐eating/purging (AN‐BP) type diagnosis according to the DSM IV (with the exception of the amenorrhea criterion)(American Psychiatric Association, 2004), and (iii) inclusion in the EVALADD cohort between September 2012 and December 2015 (to ensure at least one year of follow‐up data).

**FIGURE 1:**
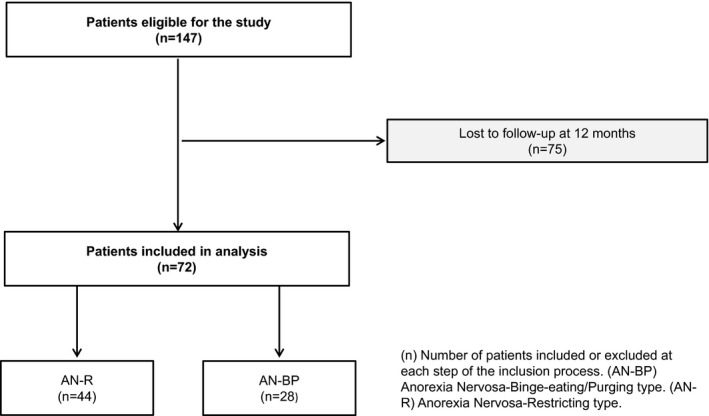
Flow chart of patient selection. (n) Number of patients included or excluded at each step of the inclusion process. (AN‐BP) Anorexia Nervosa‐Binge‐eating/Purging type. (AN‐R) Anorexia Nervosa‐Restricting type

A total of 147 patients were eligible for the study. Seventy‐five were lost to follow‐up at 1 year and consequently were not included in prognosis statistical analyses. The remaining 72 patients were included, forty‐four patients were diagnosed with AN‐R, and 28 were diagnosed with AN‐BP. The flow chart of patient selection is presented in Figure 1 (Figure 1).

### 2.3 Treatment

Our unit specializes in ED management (*i.e.,* AN, BN, and BED) and is recognized as a National Reference Center in France. To receive treatment in our unit, patients must be referred to us by a medical professional. We provide physical, psychological, and social care that is in accordance with the guidelines for ED management (Haute Autorité de Santé (HAS), 2010; National Collaborating Centre for Mental Health (UK), 2004). The care objectives of our unit are as follows: (i) to restore patients to a healthy weight, (ii) to alter core dysfunctional symptoms and attitudes related to ED (excessive concerns about body shape and weight, dietary restriction, purge and binge symptoms, etc.), and (iii) to manage all other negative features associated with ED. Treatment is primarily conducted in an outpatient format, with inpatient treatment provided only if necessary. The usual criteria for inpatient treatment are severe malnutrition, significant risk of suicide, no improvement with appropriate outpatient treatment, and severe family conflict. Treatment is adapted to patient's heterogeneity and often differs from one patient to another in accordance with ED treatment guidelines.

### 2.4 Measures

#### 2.4.1 Sociodemographic characteristics at inclusion

The sociodemographic data collected included the age and details about the patient's family support system. To assess family support, all patients were asked whether they thought that their family was supportive/helping concerning eating disorders (yes or no).

#### 2.4.2 Eating disorder diagnosis at inclusion

The fifth version of the Mini International Neuropsychiatric Interview (MINI) is a structured diagnostic interview that enables rapid and systematic investigations of the main axis 1 psychiatric disorder (Sheehan et al., 1998) according to DSM criteria (American Psychiatric Association, 2004). For the study, we used the French version of the MINI (Lecrubier et al., 1997). AN diagnosis (restricting type or binge‐eating/purging type) was made by qualified and experienced staff members according to the eating disorders (ED) sections (Anorexia Nervosa, Bulimia Nervosa, and Binge‐Eating Disorders) of the MINI with the exception of the amenorrhea criterion. The MINI enables clinicians and nonclinicians to make the diagnosis of AN according to DSM criteria which are consensus‐based criteria for AN.

#### 2.4.3 Characteristics of eating disorders at inclusion and follow‐up

##### ED‐related characteristics

To characterize the ED, a structured, standardized, and semi‐directive interview was specifically developed in order to collect data about AN (age of onset, disease duration, lowest body mass index (BMI), and current BMI), current associated behaviors (self‐induced vomiting, laxative use, problematic exercise), negative impact of AN on physical health, family life, social life, school and/or work life (using 6‐point Likert scales ranging from “no impact” to “very strong impact”), history of physical and/or sexual abuse, and past treatments for ED.

At the 12‐month follow‐up, current BMI and follow‐up ED treatment (cumulative number of days of inpatient treatment between inclusion and 12‐month follow‐up) were collected. For patients without hospitalization admission during follow‐up, the length of hospitalization was 0.

##### Body shape questionnaire (BSQ) at inclusion

The BSQ is a self‐questionnaire that was developed in 1987 by Cooper et al. (Cooper et al., 1987) and validated in French in 2005 by Rousseau et al. (Rousseau et al., 2005). This self‐assessment questionnaire explores concerns about body weight and shape, body sensation, and body‐related social interactions over the past 4 weeks. It is unidimensional and consists of 34 items with answers given on a 6‐point Likert scale ranging from “never” to “always,” resulting in an overall score between 34 and 204. The BSQ score provides a classification of the intensity of body shape concerns (< 80: “no concern”; 80 to 110: “mild concerns”; 111 to 140: “moderate concerns”; > 140: “marked concerns”)(Cooper et al., 1987; Lofrano‐Prado et al., 2011). The reliability of the BSQ has been reported to be high (α = 0.96). The BSQ has the advantage of providing a measure of body image that takes into account attitudinal and perceptual components of body image concerns (attitudes about body shape, avoidance behavior, public embarrassment, undue concerns about shape and weight).

##### Morgan and russell outcome average score (MROAS) at inclusion and follow‐up

The MROAS is a structured interview that covers various clinical symptoms of AN and their repercussions on patient functioning in the past six months (H. G. Morgan & Hayward, 1988). The questionnaire consists of five subscales exploring food intake and nutritional status, menstrual function, mental state, psychosexual adjustment, and socio‐economic status. Each subscale is scored from 1 to 12, with a higher score indicating a better outcome in the corresponding field. The average of these five scores is used as the MROAS total score, with a result ranging from 1 to 12.

#### 2.4.4 Psychiatric and addictive comorbidities at inclusion

The fifth version of the MINI (described above) was used. For the purposes of this study, only current mood disorders (major depressive episode, dysthymia, (hypo‐) manic episodes), current anxiety disorders (panic disorder, agoraphobia, social phobia, obsessive‐compulsive disorder, post‐traumatic stress disorder, generalized anxiety disorder), and current risk of suicide were considered.

#### 2.4.5 Self‐Esteem Scale (SES) at inclusion

The SES is a self‐assessment questionnaire that provides an overall evaluation of self‐esteem based on 10 items. For each of these items, the answer is provided on a 4‐point Likert scale, ranging from "strongly disagree" to "strongly agree." An overall score of less than 30 indicates low self‐esteem (Rosenberg, 1965). A validated French version of this scale was used for our study (Vallieres & Vallerand, 1990).

#### 2.4.6 Outcome measures

The primary outcome measure in this study was the MROAS “total” score at follow‐up. This choice was based on several arguments *(1)* the MROAS is a reference tool for assessing outcome in ED (Boehm et al., 2016; Lange et al., 2019; Roux et al., 2016; Winkler, 2017) *(2)* the MROAS total score not only assess core symptoms of ED but covers global functioning of patient with ED *(3)* considering the lack of insight associated with AN (Arbel et al., 2014) we promoted clinical interview rather than self‐evaluation tool. Indeed, AN patients tend to underestimate eating psychopathology (Couturier & Lock, 2006), and self‐assessment tools may not reflect the psychological state of patients (Starzomska & Tadeusiewicz, 2016; Viglione et al., 2006).

Table 1 summarizes the content of the multiaxial assessment used for the study (inclusion and follow‐up).

**TABLE 1:** Content of the multiaxial assessment used for the study (baseline and follow‐up)

Assessment	Measures
Inclusion	Informed written consent Sociodemographic characteristics ED diagnosis (MINI) Characteristics of ED ED‐related characteristics : age of onset, disease duration, lowest BMI, current BMI, current associated behaviors, negative impact of AN, history of physical and/or sexual abuse, past treatment Body Shape Questionnaire MROAS Psychiatric and addictive comorbidities (MINI) Self‐Esteem Scale
Follow‐up	Characteristics of ED ED‐related characteristics: current BMI, cumulative number of days of inpatient treatment between inclusion and 12‐month follow‐up. MROAS

Abbreviations: BMI, Body mass index; DSM‐5, Diagnostic and statistical manual of mental health disorders: 5th ed; AN, anorexia nervosa, MINI, Mini International Neuropsychiatric Interview; MROAS, Morgan and Russel Outcome Average Score.

### 2.5 Statistical analysis

Descriptive statistical analysis was conducted for the entire sample. Continuous variables were described by the mean and standard deviation, while categorical variables were presented as numbers and percentages. To assess changes over time, analyses for dependent samples were conducted for MROAS scores and BMI. We used Student's *t* tests for variables with a normal distribution and Wilcoxon nonparametric tests for variables with a non‐Gaussian distribution. A p‐value of <0.05 was regarded as statistically significant.

We divided the sample into two groups according to BSQ scores at inclusion to compare the patients with the highest body concerns to the others. The binarization of the BSQ variable allowed us to identify predictors of outcomes that could be easily identifiable and used as therapeutic targets at the beginning of treatment for ED. The first group, “No to moderate body concerns,” consisted of patients with a BSQ score of 140 or less. This group included the patients with “no concern,” “mild concerns,” and “moderate concerns” according to the BSQ classification (Cooper et al., 1987). The second group, “Marked body concerns,” consisted of patients with the highest intensity of body shape concerns, with a BSQ score higher than 140. Bivariate analyses for independent samples were conducted to explore the associations between the intensity of patients’ body concerns at inclusion and other characteristics assessed at inclusion and follow‐up. Therefore, we used Chi^2^ tests or Fisher's tests, if necessary, to analyze the qualitative variables. For the quantitative variables, we used Student's tests for variables with a normal distribution and Wilcoxon nonparametric tests for variables with a non‐Gaussian distribution.

A multiple linear regression model was used to explain the MROAS “total” score at 12 months. The variables tested in the model were the characteristics assessed at inclusion: age, type of AN, age at onset of AN, illness duration, previous medical care, MROAS “total” score, BMI, lowest BMI, BSQ score >140, laxative use, self‐induced vomiting, problematic exercise, mood disorders, anxiety disorders, suicide risk, history of physical or sexual abuse, family support, self‐esteem score, follow‐up ED treatment and impact of AN on physical health, family life, social life, and school/work life. A top–down selection process was used to find the variables significantly related to the MROAS score, and the results were adjusted for the variables.

All statistical tests were performed bilaterally with an alpha level of 5%, and p‐values less than 0.05 were considered significant. We should note that a power analysis was not conducted due to the exploratory nature of the study. All statistical analyses were carried out using R statistical software, version 3.1.2.

## 3 RESULTS

### 3.1 Representativeness of the sample

To ensure that the patients included in the study (*n* = 72) did not differ from those who were not included because they were lost to follow‐up at 12 months (*n* = 75), the variables collected at inclusion were compared. The only two significant differences were the mean age and the MROAS “Menstruation” subscale score at inclusion, which were lower in the included patients than in the patients lost to follow‐up (Table 2).

**TABLE 2:** Comparison of the patients included and the patients lost to follow‐up at 12 months (*n* = 147)

Characteristics at inclusion	Included in study (*n* = 72) *n* (%) or m (sd)	Lost to follow‐up (*n* = 75) *n* (%) or m (sd)	*p*‐value	Statistical test
Sociodemographic characteristics				
Age (year)	21.9 (7.4)	23.6 (7.0)	0.043	Wilcoxon Test
Family support	68 (94.4%)	63 (84.0%)	0.077	Chi² test
Eating disorders characteristics				
Type of AN: restricting	44 (61.1%)	46 (61.3%)	0.999	Chi² test
Age of disease onset (year)	17.6 (5.4)	16.9 (4.5)	0.669	Wilcoxon Test
Disease duration (year)	4.3 (5.1)	6.7 (7.0)	0.061	Wilcoxon Test
Lowest BMI (kg/m²)	14.2 (1.5)	14.3 (1.4)	0.513	Student test
Current BMI (kg/m²)	15.5 (1.3)	15.5 (1.5)	0.783	Student test
Body concerns (BSQ)	124.6 (33.7)	115.6 (35.3)	0.115	Student test
Current laxative use	4 (5.6%)	4 (5.3%)	0.999	Fisher test
Current self‐induced vomiting	21 (29.2%)	18 (25.3%)	0.736	Chi² test
Current problematic exercise	15 (20.8%)	14 (18.7%)	0.902	Chi² test
Previous medical care	68 (94.4%)	66 (88.0%)	0.278	Chi² test
History of physical and/or sexual abuse	6 (8.3%)	14 (18.7%)	0.113	Chi² test
Impact on physical health	3.3 (1.3)	3.6 (1.3)	0.069	Wilcoxon Test
Impact on family life	3.5 (1.2)	3.4 (1.4)	0.997	Wilcoxon Test
Impact on social life	3.3 (1.3)	3.1 (1.5)	0.567	Wilcoxon Test
Impact on schooling and/or working life	2.1 (1.3)	2.0 (1.6)	0.604	Wilcoxon Test
MROAS “total” score	5.4 (1.5)	5.6 (1.9)	0.400	Student test
MROAS “food intake”	1.1 (1.6)	1.4 (1.7)	0.268	Wilcoxon test
MROAS “menstruation”	3.4 (4.9)	4.9 (5.2)	0.045	Wilcoxon test
MROAS “mental state”	6.3 (2.3)	6.0 (2.1)	0.358	Wilcoxon test
MROAS “psychosexual functioning”	7.5 (2.5)	7.9 (2.6)	0.284	Wilcoxon test
MROAS “socio‐economic status”	8.5 (2.0)	7.8 (2.5)	0.127	Wilcoxon test
Clinical characteristics				
Current mood disorders (MINI)	24 (33.3%)	21 (28.0%)	0.601	Chi² test
Current anxiety disorders (MINI)	36 (50.0%)	42 (56.0%)	0.573	Chi² test
Current risk of suicide (MINI)	37 (51.4%)	41 (54.7%)	0.816	Chi² test
Self‐esteem (SES)	21.3 (5.6)	22.9 (5.9)	0.094	Student test

Abbreviations: %, percentage; m, mean; sd, standard deviation; AN, anorexia nervosa; BMI, Body Mass Index; BSQ, Body Shape Questionnaire; M&R, Morgan and Russel Scale; MINI, Mini International Neuropsychiatric Interview; SES, Self‐Esteem Scale.

### 3.2 Description of the complete sample

The characteristics of the complete sample are shown in Table 3. The MROAS total score and all subscales of the MROAS were significantly improved between inclusion and the 1‐year follow‐up. BMI was also significantly higher at the 1‐year follow‐up. We noted that the duration of hospitalization was generally short (on average one month), which was consistent with guidelines recommending emphasis on outpatient treatment for ED.

**TABLE 3:** Comparison of patients according to the BSQ score at inclusion (*n* = 72)

	Complete sample(*n* = 72) *n* (%) or m (sd)	“No to moderate body concerns” (*n* = 46) *n* (%) or m (sd)	“Marked body concerns” (*n* = 26) *n* (%) or m (sd)	*p*‐ value	Statistical test
Characteristics at inclusion					
Sociodemographic characteristics					
Age (year)	21.9 (7.4)	21.7 (6.9)	22.3 (8.3)	0.823	Wilcoxon Test
Family support	68 (94.4%)	43 (93.0%)	25 (96.2%)	0.999	Fisher test
Eating disorders characteristics					
Type of AN: restricting	44 (61.1%)	33 (71.7%)	11 (42.3%)	0.027	Chi² test
Age of disease onset (year)	17.6 (5.4)	17.5 (4.5)	17.8 (6.8)	0.452	Wilcoxon Test
Disease duration (year)	4.3 (5.1)	4.1 (5.4)	4.5 (4.5)	0.215	Wilcoxon Test
Lowest BMI (kg/m²)	14.2 (1.5)	14.1 (1.6)	14.2 (1.2)	0.735	Student test
Current BMI (kg/m²)	15.5 (1.3)	15.4 (1.2)	15.6 (1.3)	0.465	Student test
Current laxative use	4 (5.6%)	0 (0%)	4 (15.4%)	0.015	Fisher test
Current self‐induced vomiting	21 (29.2%)	9 (20.0%)	12 (46.2%)	0.029	Chi² test
Current problematic exercise	15 (20.8%)	9 (20.0%)	6 (23.1%)	0.960	Chi² test
Previous medical care	68 (94.4%)	46 (100%)	22 (84.6%)	0.015	Fisher test
History of physical and/or sexual abuse	6 (8.3%)	2 (4.0%)	4 (15.4%)	0.180	Fisher test
Impact on physical health	3.3 (1.3)	3.3 (1.2)	3.3 (1.4)	0.718	Wilcoxon Test
Impact on family life	3.5 (1.2)	3.4 (1.4)	3.7 (1.5)	0.048	Wilcoxon Test
Impact on social life	3.3 (1.3)	3.1 (1.3)	3.7 (1.1)	0.055	Wilcoxon Test
Impact on schooling and/or working life	2.1 (1.3)	1.8 (1.1)	2.6 (1.6)	0.019	Wilcoxon Test
MROAS “total”	5.4 (1.5)	5.7 (1.6)	4.8 (1.1)	0.023	Student test
MROAS “food intake”	1.1 (1.6)	1.5 (1.8)	0.4 (0.9)	0.006	Wilcoxon Test
MROAS “menstruation”	3.4 (4.9)	3.5 (5.0)	3.2 (4.6)	0.989	Wilcoxon Test
MROAS “mental state”	6.3 (2.3)	6.7 (2.2)	5.6 (2.3)	0.029	Wilcoxon Test
MROAS “psychosexual functioning”	7.5 (2.5)	7.9 (2.4)	6.8 (2.4)	0.061	Student test
MROAS “socio‐economic status”	8.5 (2.0)	8.6 (1.9)	8.2 (2.1)	0.367	Student test
Clinical characteristics					
Current mood disorders (MINI)	24 (33.3%)	14 (30.0%)	10 (38.5%)	0.665	Chi² test
Current anxiety disorders (MINI)	36 (50.0%)	18 (39.0%)	18 (69.2%)	0.027	Chi² test
Current risk of suicide (MINI)	37 (51.4%)	18 (39.0%)	19 (73.1%)	0.012	Chi² test
Self‐esteem (SES)	21.3 (5.6)	23.2 (5.1)	18.0 (4.9)	<0.001	Student test
Characteristics at follow‐up (12 months)					
Eating disorders characteristics					
Current BMI (kg/m²)	17.0 (2.4)*	17.3 (2.3)	16.5 (2.6)	0.232	Student test
MROAS “total”	6.8 (2.2)*	7.3 (2.1)	6.0 (2.2)	0.011	Student test
MROAS “food intake”	4.3 (3.1)*	5.1 (2.7)	2.9 (3.2)	0.002	Wilcoxon Test
MROAS “menstruation”	5.1 (5.3)*	5.6 (5.4)	4.2 (5.1)	0.331	Wilcoxon Test
MROAS “mental state”	7.5 (2.4)*	8.0 (2.3)	6.6 (2.3)	0.014	Wilcoxon Test
MROAS “psychosexual functioning”	8.2 (2.8)*	8.5 (2.9)	7.7 (5.6)	0.204	Wilcoxon Test
MROAS “socio‐economic status”	9.1 (2.2)*	9.5 (2.0)	8.4 (2.4)	0.089	Wilcoxon Test
Duration of hospitalization during follow‐up (months)	1.1 (2.0)	1.1 (2.2)	1.2 (1.6)	0.882	Student test
Evolution between inclusion and follow‐up					
Δ BMI (kg/m²)	+ 1.5 (2.1)	+ 1.9 (1.8)	+ 0.9 (2.3)	0.049	Student test
Δ MROAS “total”	+ 1.5 (2.0)	+ 1.7 (1.9)	+ 1.1 (2.0)	0.265	Student test
Δ MROAS “food intake”	+ 3.2 (2.8)	+ 3.6 (2.8)	+ 2.5 (2.9)	0.098	Student test
Δ MROAS “menstruation”	+ 1.7 (5.3)	+ 2.1 (5.4)	+ 1.1 (5.0)	0.563	Wilcoxon Test
Δ MROAS “mental state”	+ 1.2 (2.9)	+ 1.3 (2.8)	+ 1.0 (3.1)	0.696	Student test
Δ MROAS “psychosexual functioning”	+ 0.7 (2.3)	+ 0.6 (2.4)	+ 0.9 (2.3)	0.579	Student test
Δ MROAS “socio‐economic status”	+ 0.6 (2.4)	+ 0.9 (2.3)	+ 0.3 (2.5)	0.309	Student test

Abbreviations: %, percentage; m, mean; sd, standard deviation; AN, anorexia nervosa; BMI, Body Mass Index; BSQ, Body Shape Questionnaire; Δ, delta between inclusion state and follow‐up state; MROAS, Morgan and Russell Outcome Average Score; MINI, Mini International Neuropsychiatric Interview; SES: Self‐Esteem Scale.

* *p* <.05 with dependent samples analysis between inclusion and follow‐up.

### 3.3 Comparison of the patients according to the intensity of body concerns at inclusion

Of the 72 patients included in the study, 46 displayed “No to moderate body concerns” and 26 exhibited “Marked body concerns” at inclusion. Table 3 shows the results of the comparisons of the two groups.

#### 3.3.1 Comparison at inclusion

Several significant differences were observed. The “Marked body concerns” group was composed of significantly fewer AN‐R patients (42.3%) than the “No to moderate body concerns” group (71.7%). Patients from the “Marked body concern” group exhibited more self‐induced vomiting behavior and more laxative use and reported a greater impact of the illness on their lives (family, school, and/or work life). They also experienced a greater severity of the illness, as demonstrated by the MROAS scores at inclusion (lower MROAS “total” score, “Food intake,” and “Mental state” scores). Except for current mood disorders, comorbidities were more prevalent in the “Marked body concerns” group (current anxiety disorders and current risk of suicide), and self‐esteem was worse. Patients from this group were less likely to have received prior medical care for their AN than the group with “No to moderate body concerns.”

#### 3.3.2 Comparison at follow‐up

The “Marked body concerns” group showed a greater illness severity at the 12‐month follow‐up, as demonstrated by the MROAS scores at 12 months (lower MROAS “total” score, “Food intake,” and “Mental state” scores).

#### 3.3.3 Evolution between inclusion and follow‐up

No difference in the MROAS scale was noted. The only difference when we compared the evolution of the two groups was the increase in BMI, which was significantly greater in the “No to moderate body concerns” group, with a gain of 1.9 points after 12 months (*SD*=1.8), than in the “Marked body concerns” group.

### 3.4 Factors associated with the AN severity at 12 months

Table 4 presents the variables found to be significantly associated with the MROAS “total” score at 12 months in the multiple linear regression model. Patients with marked body concerns showed an MROAS “total” score at 12 months that was 1.453 points lower on average than those of patients with no to moderate body concerns, regardless of the variables introduced in the model (including the MROAS “total” score at inclusion). The MROAS “total” score at inclusion was also found to be linked to the MROAS “total” score at 12 months, regardless of the other variables.

**TABLE 4:** Multiple linear model with variables significantly associated with the Morgan and Russell “total” score at follow‐up (*n* = 72)

Model	Coefficient	Standard Error	*p*‐value	CI 95%
BSQ score >140	−1.453	0.457	0.002	[−2.365; −0.540]
MROAS “total” score at inclusion	0.437	0.144	0.003	[0.150; 0.724]
Duration of hospitalization during follow‐up (months)	−0.400	0.106	<0.001	[−0.612; −0.189]
Impact on schooling and/or working life				
5—very strong impact	1.964	0.643	<0.001	[0.681; 3.248]

Abbreviations: BMI, Body Mass Index; BSQ, Body Shape Questionnaire; CI, Confidence Interval; MROAS, Morgan and Russell Outcome Average Score.

Treatment for ED during follow‐up was significantly associated with clinical outcomes at 12 months, and the cumulative number of days of inpatient treatment was inversely correlated with the MROAS score at 12 months. Finally, reporting a “major” (score=5) impact of AN on school and/or work life was significantly associated with clinical improvement, as indicated by an average gain of nearly 2 points in the MROAS “total” score at 12 months, compared with the scores of patients who reported no impact of AN.

## 4 DISCUSSION

The overall evolution of the patients at the 12‐month follow‐up was favorable, regardless of the intensity of their body concerns at inclusion. When we considered the whole sample of patients, BMI and all MROAS scores were significantly improved at the 12‐month follow‐up. Between inclusion and follow‐up, we observed increases in BMI (+1.5 on average) and for the MROAS “total” score (+1.5 on average between inclusion and follow‐up), with the strongest evolution for the food intake subscale. The latter increase could be interpreted as low, but it represents an evolution of nearly 30% of the MROAS “total score” between inclusion and follow‐up. The slight improvement is not a surprise because we know that AN is a long‐term disease that evolves over several years (Haute Autorité de Santé (HAS), 2010; Strober et al., 1997). Epidemiological studies on the AN course showed a 5‐year clinical recovery rate of nearly 67% (Keski‐Rahkonen et al., 2007). In studies with a follow‐up of 1 year to 4 years, the recovery rate was only 30%, which was equal to the chronicity rate (Steinhausen, 2002). The 2019 German guidelines noticed that a quarter of adult patients go on to develop an enduring form of AN, and one‐third of patients continue to suffer from residual symptoms in the long term (Resmark et al., 2019). To interpret the results, we must also consider that patients referred to our department, which is a tertiary care center specializing in ED treatment, are usually more severe, and consequently, clinical improvement could be longer to achieve.

The bivariate comparison of patients with or without marked body concerns showed a significant association between the intensity of body concerns and the severity of clinical characteristics at inclusion, as shown previously in a study by Gailledrat and colleagues (Gailledrat et al., 2016). Indeed, the characteristics at inclusion showed that the MROAS “total” score was lower for patients with high body concerns. These latter were more frequent in the AN binge‐eating/ purging type with more laxative use and self‐induced vomiting behaviors. These patients exhibited more anxiety disorders, greater risk of suicide, and a lower self‐esteem. It can be hypothesized that a lack of feelings, as well as rigid self‐control over emotions, has a protective effect against anxiety and depression in patients with AN‐R (more frequent in the “No to moderate body concerns” group). Indeed, their high level of self‐control allows them to maintain their self‐esteem, whereas patients with purge behaviors are most often affected by feelings of shame and guilt. Pervasive thoughts about body concerns, as well as the negative emotions (anxiety, sadness) that they may cause, seem to be consistent with these results and reflect the poorer clinical course for patients with marked body concerns.

At 12 months, the MROAS subscale scores of patients with higher body concerns were lower in terms of “Food intake” and “Mental status.” When we focused on the evolution characteristics between inclusion and 12 months, the BMI is the only variable that evolved significantly less for the marked body concerns group. We can assume that having fewer concerns could facilitate weight gain. Indeed, a higher level of shape concern is associated with a lack of response to treatment (Ricca et al., 2010), and patients who overestimate their own body size achieve less weight gain during treatment (Sala et al., 2012).

The multivariate analysis confirmed that the intensity of body concerns is closely related to patients’ clinical evolution, as evaluated by the MROAS scale. Indeed, our results showed that having a high level of body concerns at inclusion was a factor associated with a less favorable outcome at 12 months, regardless of all other variables (including the MROAS “total” score at inclusion, the type of AN, the age of AN onset, the illness duration, and the history of previous medical care). Other studies have already highlighted the negative prognostic impact of body concerns (Boehm et al., 2016; Cash & Deagle, 1997; Vall & Wade, 2015; Warah, 1989). Our results were consistent with previous findings and contribute to the existing literature on AN outcome in the framework of an outpatient treatment. Thus, the presence of a high level of body concerns could be an important moderator in the outcome of treatment, throughout the course of the disease (Ricca et al., 2010; Strober et al., 1997).

The importance of focusing on prevention and treatment interventions in the management of body image disorders has been highlighted before (Stice & Shaw, 2002), and the results from recent specific interventions focused on body concerns are promising (Aspen et al., 2015; Jansen et al., 2016; Keizer et al., 2019; Legenbauer et al., 2011; J. F. Morgan et al., 2014). Regarding the other variables associated with evolution of the MROAS total score at 12 months in the multivariate analysis, it is interesting to note that the cumulative number of days of inpatient treatment during follow‐up was inversely correlated with the MROAS score at 12 months. We can hypothesize that patients with longer inpatient treatment were more severe than the other patients and have evolved to a lower extent. In addition, we also showed a lower MROAS “total” score at inclusion (*i.e.,* patients with more severe ED at inclusion) was significantly associated with poorer outcome at the 12‐month follow‐up. The impact on school and/or work life also seemed to play a positive role in the clinical course; we can assume that awareness of the consequences of their disorders in these domains could motivate these patients to change their lifestyle. Patients with AN often do not consider the manifestations of their illness as a source of concern (Treasure, 2016), or they deny them (Couturier & Lock, 2006). As a consequence, ambivalence toward treatment is typical in AN (Williams & Reid, 2010). Enhancing motivation to change is thus a challenge for clinicians (Casasnovas et al., 2007), and there is little evidence that current models used to induce motivation to change in AN patients are effective (Waller, 2012). To our knowledge, the impact of AN on schooling and/or working has never been associated with AN outcomes before, and our results suggest that this area could be a good target for motivational interviews.

To conduct our study, we chose to include patients with a diagnosis of AN of the restricting or purging type only and excluded patients suffering from bulimia nervosa or EDNOS in order to limit the biases related to differences in the underlying psychopathological manifestations of the various EDs. For the same reason, we chose to include only women. These inclusion criteria allowed us to obtain a homogeneous sample of patients; however, the results cannot be extrapolated to the rest of the population. In this follow‐up study, a significant number of patients were lost to follow‐up; however, after comparing the data from the patients lost to follow‐up with those of the included patients, we found a significant difference in age: Patients who were lost to follow‐up were significantly older. This difference could be explained by the fact that older patients were less often accompanied by their family (especially their parents) and therefore experienced more challenges in continuing their follow‐up. This result is in line with the work of Godart and colleagues, who reported that being over 18 at admission to an inpatient treatment was a predictor of dropout (Roux et al., 2016). The main bias in our study is a declarative bias. Indeed, this study was based partly on responses to self‐reported questionnaires, and we acknowledge that denial is usually an important characteristic of ED patients. In addition, considering bias associated with denial, especially with self‐evaluation tools, we chose to use a clinical interview for our primary outcome measure (the MROAS). Finally, due to the relatively limited sample size applied, the analysis probably lacks power, potentially leading to the incorrect conclusion that certain variables did not play any role in outcome.

## 5 CONCLUSION

Our study confirms the importance of investigating the intensity of body concerns in ED patients. Assessing these factors will allow a better understanding of these patients’ body issues, which will help ensure that they receive the most appropriate treatment. Characteristics at inclusion that were predictive of a better outcome at 12 months were having less severe ED at inclusion, having no or moderate body concerns, having a shorter inpatient setting during follow‐up, and experiencing a greater impact of the illness on school/work life. These results confirmed the importance of a multifocal treatment that should address body concerns and motivation to change. Our results also highlighted the necessity of promoting the maintenance of school/work insertion during treatment. It would be interesting to extend this research over a longer follow‐up period and to include repeated measures of body concerns to analyze the longer‐term outcomes of the patients and to assess how the evolution of body concerns during follow‐up could influence the outcome of the illness.

## CONFLICT OF INTEREST

All authors declare that they have no conflicts of interest.

### PEER REVIEW

The peer review history for this article is available at https://publons.com/publon/10.1002/brb3.2199.

## Data Availability

All participants provided written informed consent (for those under 18 years old, written informed consent was provided by a legal representative) for using data for this study with data processing and storage in secure database from Nantes University hospital. They did not provided nonopposition for the use of data for furthers researches. However, we are willing to make our data available upon request as we consider that it is important for open and reproducible science, and thus, we will ensure that all interested and qualified researchers will be able to be granted access.
